# Taxonomic and transcriptional associations between arbuscular mycorrhizal fungi and the soil microbiome are maintained under biotic perturbation

**DOI:** 10.1093/ismeco/ycag163

**Published:** 2026-06-11

**Authors:** Fergus Wright, Ian R Sanders, Ricardo Arraiano-Castilho

**Affiliations:** Department of Ecology and Evolution, University of Lausanne, Lausanne 1015, Switzerland; Department of Ecology and Evolution, University of Lausanne, Lausanne 1015, Switzerland; Department of Ecology and Evolution, University of Lausanne, Lausanne 1015, Switzerland

**Keywords:** soil microbiome, perturbation, AMF, *Rhizophagus irregularis*, microbial interactions, metatranscriptomics

## Abstract

Arbuscular mycorrhizal fungi (AMF) structure soil microbiomes and support plant growth. However, the stability of established AMF-microbiome interactions to biotic perturbation by introduced microbial inocula is unclear. We investigated how AMF shape soil microbial communities and transcriptional activity when challenged by the introduction of foreign microbial inocula. Using established maize-AMF mesocosms, we introduced microbial communities from forest and agricultural soils and assessed taxonomic composition and metatranscriptomic profiles. Despite clear differences between introduced inocula, neither microbiome treatment altered resident microbial community composition or transcriptional profiles. Instead, AMF exerted a strong and consistent influence on microbial gene expression and bacterial taxa abundances, with effects scaling quantitatively with the degree of mycorrhizal colonization. Co-expression analyses revealed coordinated transcription among plant, AMF, and microbial genes, suggesting multi-kingdom interactions. Overall, we find AMF drive the structure of soil microbial communities, with biotic perturbation exerting limited influence under the conditions tested.

## Introduction

The development of microbial inocula for agriculture and ecosystem services requires understanding how introduced organisms interact with established soil microbiomes. Arbuscular mycorrhizal fungi (AMF) are keystone soil taxa that benefit plant nutrient acquisition, soil structure, nutrient cycling and plant diversity [1]. Through hyphal exudation and resource redistribution, established AMF-plant systems shape microbial assemblages in both the rhizosphere and bulk soil [2, 3]. These AMF-associated microbial communities promote host plant fitness, for example supressing pathogens through niche occupation and competitive exclusion [4, 5]. Despite increasing recognition of AMF-mediated microbiome structuring [3, 6], the stability of established AMF-microbiome interactions to introduced foreign microbial communities is unknown; in particular, the resistance (capacity to remain unchanged after perturbation) and resilience (capacity to recover following the perturbation) as defined in ecosystem research [7, 8].

Here, we investigated the response of characterized plant-AMF-microbiomes [3] to perturbation by two taxonomically and functionally distinct microbial inocula from forest (F) and agricultural (A) soils. Existing plant-AMF-microbiome mesocosms contained one of two different genotypes of the AMF *Rhizophagus irregularis* in association with an established microbial community [3] and allowed for sampling of the rhizosphere and bulk soil. We tested three hypotheses: (i) introduction of A or F inocula perturbs the existing microbiome, resulting in shifts in taxonomic composition and gene transcription across rhizosphere and bulk soil; (ii) microbial gene transcription is associated with microbial taxa added in the A and F treatments; (iii) rhizosphere microbial gene transcription is correlated with maize and AMF gene transcription, representing functional inter-kingdom interactions.

The experiment was conducted in a greenhouse with mesocosms containing 70-day old maize plants, in symbiosis or not, with one of two *R. irregularis* genotypes (C2 and DAOM197198). Following substrate sterilization (Supplementary note S1), mesocosms developed a resident microbial community derived from the greenhouse environment [3], which established for 70 days prior to perturbation with the A or F inocula (Supplementary note S2). Shotgun metatranscriptomics of the rhizosphere and bulk soil was conducted, and short-read Illumina amplicon sequencing performed (Supplementary note S3). Detailed bioinformatic and statistical methods are provided in Supplementary notes S4 and S5.

## Results and discussion

**Figure 1:**
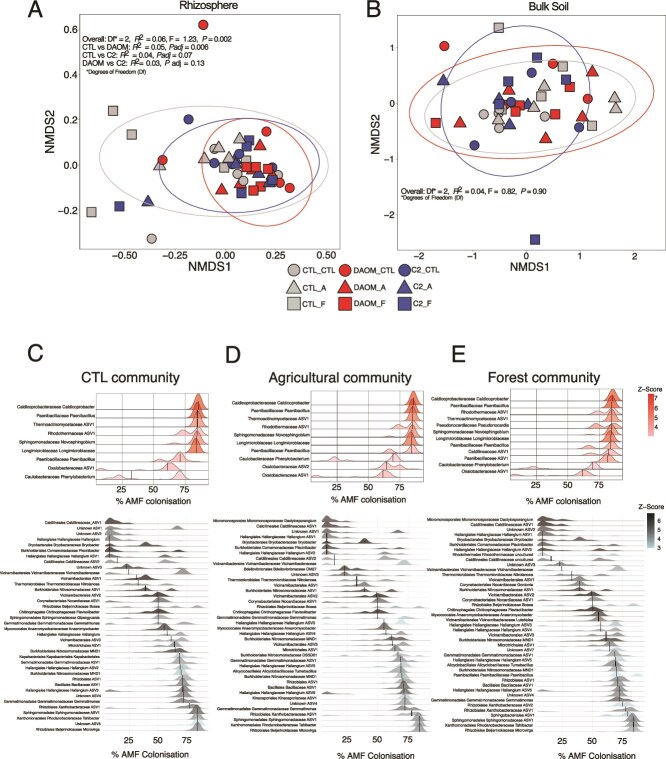
(A & B) Effects of microbial inocula and AMF genotype on the composition of transcribed genes in the rhizosphere and bulk soil. Colours represent AMF inoculation treatments: Grey not inoculated with AMF; red inoculated with DAOM; and blue inoculated with C2. Symbols represent microbial inoculations treatments: Circles not inoculated; triangles inoculated with a inoculum; squares inoculated with F inoculum. (A) Bray-Curtis NMDS ordination of UniProt annotated mRNA transcripts from the rhizosphere. PERMANOVA analysis showed that AMF significantly affected mRNA transcript composition (R^2^ = 0.055, *F* = 1.23, *P* = .002), whereas the A & F inoculants (R^2^ = 0.042, *F* = 0.94, *P* = .884) and the interaction between AMF and the a&F inoculants (R^2^ = 0.094, *F* = 1.04, *P* = .143) were not significant. Post hoc analysis found that CTL and DAOM treatments were significantly different from each other, the overall model and post hoc comparisons are listed on the top right of the plot. (B) Bray-Curtis NMDS ordination of UniProt annotated mRNA transcripts in the bulk soil. No significant differences were found between AMF treatments, microbial inoculants or the respective interaction term. (C), (D), and (E) Indicator taxa associated quantitatively with the amount of AMF colonization across microbial inoculation treatments in the rhizosphere based on 16S sequence data. Red colour represents positively associated taxa, and blue colour represents negatively associated taxa. (C) Positive and negative associations between bacterial taxa in CTL treatments and different levels of AMF root colonization. (D) Positive and negative associations between bacterial taxa in agricultural inocula treatments and different levels of AMF root colonization. (E) Positive and negative associations between bacterial taxa in forest inocula treatments and different levels of AMF root colonization. A higher Z-score is represented by darker shading and indicates a stronger and more consistent threshold response of the specific taxon along the AMF colonization gradient compared to random chance.

Fungal and bacterial taxonomic composition between soil-wash derived A and F microbial communities was distinct (Supplementary Fig. S1). However, 30 days after inoculation, we observed no significant differences in bacterial and fungal taxonomic composition in the rhizosphere or bulk soil compared to non-inoculated controls (Supplementary Figs S2 and S3). Furthermore, introduced inocula did not significantly alter the composition of transcribed microbial genes between treatments in either rhizosphere (Fig. 1A) or the bulk soil (Fig. 1B). The pre-existing microbial community had likely already occupied all niches, affording resistance to establishment of introduced A and F inocula [9] or habitat filtering [10] meant the introduced inoculum poorly adapted to mesocosm conditions compared to the established community. We also cannot rule-out the inoculum dosage was insufficient to cause a shift in community composition even though the dosage used was in line with previous research [10].

The *R. irregularis* DAOM197198 significantly altered the composition of soil microbial transcripts compared to no AMF inoculum controls (herein CTL) treatment (Fig. 1A) in the rhizosphere, irrespective of the microbial inocula treatments. This is in contrast to our previous work which found an effect in the bulk soil not the rhizosphere at an earlier timepoint [3] implying both temporal and spatial dynamics are important in AMF-microbe interactions. Several bacterial taxa were significantly associated, either positively or negatively, with AMF according to the level of AMF colonization (Fig. 1  C, D, and  E). These quantitative relationships between bacterial taxa abundances and AMF colonization were remarkably consistent across treatments, despite potential perturbation by added microbes (Fig. 1  C, D, and  E). *Oxalobacteraceae* [11] and *Paenibacillaceae* [12, 13] have previously been found positively associated with AMF whereas abundances of *Sphingomonadales* and *Rhizobiales* have previously been found negatively correlated with AMF [14]. However, most other studies focused on bacterial taxa abundances that are affected by the presence or absence of AMF. Here, our findings show the quantitative effects of increasing or decreasing amounts of AMF and how they consistently shape abundances of many bacterial taxa.

**Figure 2:**
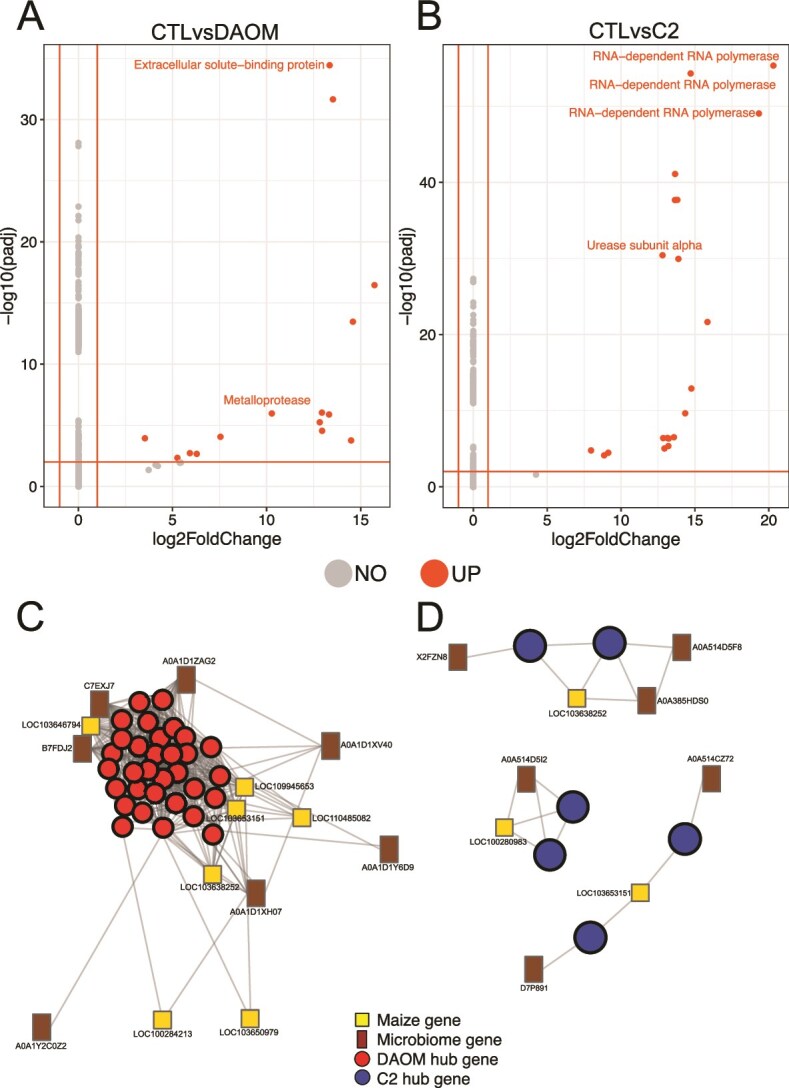
The effect of DAOM and C2 on transcribed genes in the rhizosphere (A) volcano plot of differentially expressed transcribed genes in the rhizosphere in DAOM treatments versus non-inoculated treatments. (B) Volcano plot of differentially expressed transcribed genes in the rhizosphere in C2 treatments versus non-inoculated treatments. In both A & B, differentially transcribed genes mentioned in the text are labelled with their UniProt-derived protein names. The remaining genes can be found in Supplementary data 1. (C & D) Co-occurrence network analysis based on gene transcription to determine associations among AMF genes and differentially transcribed genes from the microbiome and maize in the presence of DAOM (C) and C2 (D) in the rhizosphere. Only clusters of genes containing AMF, microbiome and maize genes are plotted, and within these clusters only AMF hub genes (defined as AMF genes within the top 10% of connectivity based on their interactions with maize and microbiome genes in the network) are displayed.

Next, we sought to identify potential microbial genes relevant to the AMF symbiosis and how they relate to plant and AMF gene expression. Fifteen and 20 soil microbial genes were significantly upregulated in DAOM197198 and C2 treatments, respectively, compared to non-inoculated treatments (Fig. 2A and B). No genes were significantly downregulated. Most genes were related to bacterial growth and metabolism (Supplementary data 1). In the DAOM197198 treatment, upregulation of *Extracellular solute-binding protein* and *Metalloprotease* may suggest that organic molecules and ions are being exchanged with the associated microbiome [15, 16]. In C2 inoculated treatments, upregulation of *Urease alpha subunit* associated with bacterial nitrogen metabolism [17], could be related to AMF preferentially assimilating ammonium [1]. Three *RNA-dependent RNA polymerases* that aligned to sequences from fungal mycoviruses (*Mitoviridae*) were also upregulated (Fig. 2B). One mycovirus was previously isolated from C2 that we used in this experiment [18]. However, the role these viruses play in AMF or fungal biology remains unclear [19].

Multi-partite co-occurrence gene expression networks occurred among AMF, maize and microbial genes (Fig. 2  C, D and Supplementary data 2), suggesting functional interdependencies between bacteria, AMF and plant gene expression. In both DAOM and C2 treatments, the maize gene *senescence-specific cysteine protease SAG39 (LOC103638252)* was clustered alongside AMF hub and soil microbial genes (Fig. 2  C and D). This gene is involved in controlled cell death [20]. We have previously reported that it is induced under AMF colonization [3]. In C2 treatments, this AMF-induced gene also occurred in the same cluster as a transcript derived from mycoviruses, strengthening evidence for multi-kingdom interdependencies (Fig. 2D). Unfortunately, all identified AMF hub genes remain uncharacterised making it impossible to determine the functional role of AMF in these gene co-expression networks.

## Conclusion

AMF consistently structured rhizosphere microbiomes at taxonomic and transcriptional levels, enriching specific bacterial taxa in a way that is shaped, not only by the presence of AMF, but by the percentage of AMF colonization. Once functional associations were established, AMF-associated communities were taxonomically and functionally stable in response to introduced foreign microbes. This highlights the ecological significance of AMF in stabilizing microbiome assembly and function.

## Supplementary Material

Supplementary_material_ycag163

## Data Availability

Amplicon and metatranscriptomic raw sequence data can be found in the NCBI Sequence Read Archive (SRA) under project number PRJNA1423709.
