# Supplementary material for: Taxonomic and transcriptional associations between arbuscular mycorrhizal fungi and the soil microbiome are maintained under biotic perturbation
Source: ISME Commun. 2026 Jun 11;6(1):ycag163. doi: 10.1093/ismeco/ycag163 (PMC13362961; doi:10.1093/ismeco/ycag163)
Supplement: Supplementary_material_ycag163 [file supplementary_material_ycag163.zip › c2_supp_info_final_2.docx]

**Supplementary Information**

**Title**

Taxonomic and transcriptional associations between arbuscular mycorrhizal fungi and the soil microbiome are maintained under biotic perturbation

**Short running title**

AMF-soil microbiome interactions

**Authors**

Fergus Wright, Ian R. Sanders^¶^ & Ricardo Arraiano-Castilho^¶^

**Authors affiliations**

Department of Ecology and Evolution, University of Lausanne, Lausanne 1015, Switzerland

^¶^ These authors contributed equally as senior authors of this work.

**Correspondence**

Ricardo Arraiano Castilho, Department of Ecology and Evolution, University of Lausanne, Lausanne, Vaud, 1015, Switzerland

E-mail: [ricardo.m.castilho@gmail.com](mailto:ricardo.m.castilho@gmail.com)

**ORCID Ids**

Fergus Wright – <https://orcid.org/0009-0009-2050-0436>

Ian Sanders – <https://orcid.org/0000-0002-9591-8214>

Ricardo Arraiano Castilho - <https://orcid.org/0000-0001-8465-5909>

**Supplementary notes S1 - Detailed information on plant, fungal, substrate and potting material**

**Plant material**

Surface sterilisation of seeds of *Zea mays* (B73) was performed and the seeds were left on moistened filter paper in petri dishes to germinate in the dark at 25 degrees for 4 days. These seeds had received no prior seed treatment or coating. The surface sterilisation procedure was as follows. Ethanol (70%) was used to initially clean seeds by gently stirring for 3 minutes. The ethanol was decanted and the seeds were washed with 7% sodium hypochlorite (approximately 1 mL per seed) for 15 min. After washing with sodium hypochlorite, the seeds were washed in sterile water for two minutes five times. They were then soaked in sterile water for 12 h. These surface sterilised seeds were germinated in glass petri dishes containing autoclaved filter paper with 2ml of water pipetted on top to maintain humidity. Typical germination time for seeds was 48h and after 3 days they were ready to be transplanted.

**Substrate**

The substrate was a 3:1 mixture of clay loam topsoil from a construction site located at the University of Lausanne with 1.7mm quartz sand. The soil had previously been twice sterilised using a Sterilo machine (Harter ERDDÄMPFER, <https://sterilo.de>). The internal temperature of the soil (centre of the Sterilo machine) was raised to 110 degrees for 90 minutes and mixed between the runs to ensure homogenous sterilisation. The sand had previously been sterilised once at 110 degrees for 90 minutes using the Sterilo machine. The resultant substrate had the following chemical properties; pH 8.0, Inorganic C [%] 0.5, Organic C [%] 1.1, Total N [%] 1.1, C/N ratio 4.7, Exch Al [cmol/kg] 0.01, Exch Ca [cmol/kg] 18.66, Exch K [cmol/kg] 0.7, Exch Mg [cmol/kg] 0.4, Exch Na [cmol/kg] 0.2, Exch P [cmol/kg] 0.06, CEC [cmolc/kg] 39.1, Base Saturation [%] 99.9, Al tot [mg/kg soil] 7453, Ca tot [mg/kg soil] 11973, Cu tot [mg/kg soil] 10, Fe tot [mg/kg soil] 6558, K tot [mg/kg soil] 1947, Mg tot [mg/kg soil] 2238, Mn tot [mg/kg soil] 222, Na tot [mg/kg soil] 255, P tot [mg/kg soil] 296, Si tot [mg/kg soil] 227, Zn tot [mg/kg soil] 25.

**Creation of pot mesocosms with rhizosphere and bulk soil**

Forty-five twelve litre pots were filled with substrate and 2 modified falcon tubes, filled with the same substrate, were inserted in each pot. This represented 5 replicates per AMF in combination with soil inoculation treatment. At the centre of each pot one sterilised and pregerminated *Z. mays* seed was transplanted, and the pots were placed in a randomised block design in the greenhouse at 28 degrees with 60% relative humidity and a 12-hour photoperiod. Pots were watered twice a week with 500ml of tap water. The two modified falcon tubes in each pot represent a rhizosphere compartment and a bulk soil compartment. The rhizosphere compartment allowed access to plant roots, AMF hyphae and the soil microbiome. It was created by cutting a hole along one side of a falcon tube. The bulk soil compartment was the same as the rhizosphere compartment, but the hole was covered with mesh (<32µm) that prevented plant root access but allowed access by AMF hyphae and the soil microbiome.

**Fungal material**

Spores of *Rhizophagus irregularis* DAOM 197198 were provided in suspension by a commercial producer (Symbiom, Sázava 170, 56301 Lanškroun, Czech Republic) having been cultured in vitro in root organ cultures. Spores of *R. irregularis* C2 were produced from our own lab collection using root organ cultures and prepared as spore suspensions for inoculation of plants. Approximately 500 spores of each AMF isolate were used to inoculate *Zea mays* plants in AMF treatments. The spore suspension was pipetted evenly around the base of *Zea mays* plants seven days after they had been planted. Controls were inoculated in the same way but using only sterile Mili-Q water.

**Supplementary notes S2 – Full details on preparation of biotic perturbation and harvest of experiment**

**Biotic perturbation**

After 70 days of maize growth, when both the plant and AMF hyphae had grown throughout the pot, a microbial perturbation was added to the mesocosm. This consisted of two different soil microbial communities prepared as follows. Agricultural and forest soil samples were collected from two sites near the University of Lausanne (46.520091, 6.575170 - agricultural soil, 46.522327, 6.577736 – forest soil) from herein referred to as A and F respectively. The agricultural site had been planted in mixed rotation for the past 10 years and at the time of sampling was planted with maize. The forest site was mixed woodland with both deciduous and coniferous species present. The selection of these two soil sites aimed to maximise the taxonomic and functionally differences between the two communities that were being added to the experiment.

Soil inoculations were prepared based on a previously published protocol (Wagner et al., 2014). To prepare the inocula both soil A and F were passed through a kitchen sieve to remove large stones and debris and 200g of the sieved soil was weighed into beakers. 1l of MES solution was added to the beakers and the soil solutions were stirred for 30 minutes. After 30 minutes of stirring the solutions were left to settle for 5 mins before being passed through a 32-micron sieve into new beakers. The sieved soil solution for both A and F were poured into 50ml centrifuge tubes and spun for 30 minutes at 4000rpm on a centrifuge. The supernatant was poured-off and the remaining pellet was resuspended in fresh MES solution. In total this produced 1l of inoculum for each soil type. 20ml of the inoculum was mixed with 500ml of water and added to each pot depending on the treatment (15 pots for A and 15 pots for F). A further 15 pots were inoculated with 20ml MES solution and 500ml water as controls. 100ml of both A and F inoculum was reserved for amplicon sequencing of the microbial communities (Supplementary Fig. S3).

Thirty days after the inoculums had been added the rhizosphere and bulk soil compartments were removed and immediately placed in liquid nitrogen for later shotgun metatranscriptomic and amplicon sequencing. One centimetre root fragments were also sampled from the entire rhizosphere and stored in 3-parts ethanol 1-part acetic acid for later root staining.

**Root staining**

An adapted protocol based on the ink-vinegar method was used to stain the roots (Vierheilig et al., 1998) whereby roots were cleaned with a 10% KOH solution, and stained with an ink-vinegar solution (1-part Pelikan 4001 Blue-Black ink, 19-parts 5% acetic acid solution). To assess the level of colonisation, 30 1cm fragments of stained roots were placed on one microscope slide. For each replicate three microscope slides were prepared. Average colonisation was calculated by assessing the presence/absence of mycorrhizae in each of the 1cm root fragments, giving 90 binary measurements per treatment.

**Supplementary notes S3 – Full details about soil nucleic acids isolation, library preparation and sequencing**

**Shotgun metatranscriptomic sequencing of rhizosphere and bulk soil**

Both the rhizosphere and bulk soil were sampled for metatranscriptomic sequencing as follows. For each AMF treatment (CTL, DAOM, C2) there were 3 soil inoculations (CTL, A, F) representing 9 treatment combinations. 5 replicates of each treatment combination were sequenced for the rhizosphere and bulk soil separately, giving a total of 45 extractions from rhizosphere compartments and 45 from bulk soil compartments. Soil total RNA was isolated using the RNA purification Kit (Norgen Biotek, Thorold, Canada) following the standard protocol with the addition of a genomic DNA removal step.

Metatranscriptomic library preparation and sequencing were conducted at the Genomic Technologies Facility (GTF) at the University of Lausanne. Sequencing libraries were prepared differently depending on the soil types. For the rhizosphere, both bacterial ribosomal RNA (rRNA) and plant rRNA depletion kits were used (Qiagen QIAseq FastSelect – 5S/16S/23S Kit and Qiagen QIAseq FastSelect –rRNA Plant Kit). For the bulk soil, only bacterial rRNA depletion was conducted using the Qiagen QIAseq FastSelect –5S/16S/23S Kit. Paired-end sequencing (150bp) was conducted using an Illumina NovoSeq 6000 sequencing platform.

**Amplicon sequencing of rhizosphere and bulk soil**

Both the rhizosphere and bulk soil were sampled for amplicon sequencing from the same mesocosms and in the same way as described above for the metatranscriptomic sequencing, with 45 extractions from rhizosphere compartments and 45 extractions from bulk soil compartments. Soil total DNA was isolated using the DNeasy PowerSoil Pro Kit (Qiagen, Hilden, Germany) following the standard protocol. Library preparation and sequencing were conducted at Génome Québec (Montréal, Canada) using Illumina MiSeq v3, PE-250 bp platform following their metabarcoding (16S, 18S, ITS) procedure. For fungi we targeted the ITS2 region using the primer pair fITS7-ITS4 (Ihrmark et al., 2012; White et al., 1990) and for bacteria we targeted the V3-V4 region of the 16S rRNA gene using the primer pair 347F-803R (Nossa, 2010). All PCRs were performed in three replicates per sample (25ul each) and pooled before library preparation using 0.02 U/ul FastStart High Fi 5U-ul (Roche), 1X PCR Buffer with 18 mM MgCl_2_ (Roche), 5% Dimethyl Sulfoxide (Roche), 0.2 mM Deoxynucleotide (dNTP) Solution Mix 10mM (New England Biolabs), 0.6 uM of each primer pair and final volume adjusted with ultra pure H_2_O. Thermal cycling conditions for the ITS2 region (fITS7-ITS4) included an initial denaturation 96 °C for 15 min; 33 cycles of denaturation for 30s at 96°C, annealing for 30s at 52°C, elongation for 1min at 72°C; final elongation at 72 °C for 10 min. For the V3-V4 region of the 16S rRNA gene (347F-803R), an initial denaturation 94 °C for 2 min; 26 cycles of denaturation for 30s at 94°C, annealing for 30s at 55°C, elongation for 1min at 72°C; final elongation at 72 °C for 10 min. Indexing of each PCR pool was conducted using 0.025 U/ul FastStart High Fi 5U-ul (Roche), 1X PCR Buffer without MgCl_2_ (Roche), 1.8mM of MgCl_2_ (Roche), 5% Dimethyl Sulfoxide (Roche), 0.2 mM Deoxynucleotide (dNTP) Solution Mix 10mM (New England Biolabs), 2 ul of each index at 2 uM and final volume adjusted to 20ul with ultra pure H_2_O. Thermal cycling conditions for indexing were: initial denaturation 95 °C for 10 min; 15 cycles of denaturation for 15s at 95°C, annealing for 30s at 60°C, elongation for 1min at 72°C; final elongation at 72 °C for 3 min for both regions. All PCR products were puryfied with sparQ PureMag Beads (Quantabio) following the manufacture’s protocol.

**Supplementary notes S4 – Detailed overview of the bioinformatics pipeline used to process the raw reads from the metatranscriptome and amplicon sequencing data**

**Analysis of soil metatranscriptome**

The quality of raw sequence reads was checked using FastQC (v0.11.9). Paired-end reads were merged using PEAR (v0.9.8) (Zhang et al., 2014) to extend contigs to approximately 250bp. SortMeRNA (v4.0) (Kopylova et al., 2012) was used to sort the merged contigs into rRNA sequences and non-rRNA sequences using the SILVA SSU and LSU databases (both v138.1) as well as UNITE fungal ITS database (vUNITE_public_all_10.05.2021). Diamond (version 2.0.15) (Buchfink et al., 2015) alignment software was used to align the unaligned short reads produced by SortMeRNA for each sample to the UniProtref90 database (v2023_01). A 90% sequence similarity cut-off was set to filter for sequences most likely to be true matches.

**Analysis of amplicon sequencing data**

Sequences were quality filtered using bbtools v39.01 (https://sourceforge.net/projects/bbmap/), adapter trimmed using cutadapt v4.1 (Martin, 2011) and denoised into amplicon sequence variants (ASVs) using dada2 v1.24 (Callahan et al., 2016) for the 16S rRNA amplicon or clustered at 97% similarity into operational taxonomic units (OTUs) for fungi. Taxonomic classifications were assigned to features using a Bayes classifier fitted with trained models on the target amplicon regions of the SILVA v138.1 (Glöckner et al., 2017; Quast et al., 2012; Yilmaz et al., 2014) and UNITE v9 (dynamic all eukaryotes 25.07.2023) (Abarenkov et al., 2024) databases for bacteria and fungi, respectively. All steps were implemented using qiime2 pipeline v2022.8.0 (Bolyen et al., 2019) with feature count tables and taxonomic maps exported to phyloseq v1.38 (McMurdie & Holmes, 2013) for downstream analysis.

**Analysis of transcriptomic responses in *Zea mays* and *R. Irregularis***

For *Zea mays* STAR (v2.7.6a) aligner was used to align all non-rRNA reads from the rhizosphere compartment to the *Zea mays* B73 reference genome (v4) (Dobin et al., 2013; Jiao et al., 2017). Similarly for *R. irregularis*, STAR aligner was used to align all non-rRNA reads from the rhizosphere and bulk soil compartment to the *R. irregularis* DAOM 197198 reference genome (NCBI accession GCF_000439145.1). In both cases BUSCO (Manni et al., 2021) was used to check transcriptome completeness and featureCounts (v2.0.3) (Liao et al., 2014) to create count tables.

**Supplementary notes S5 – Detailed overview of all statistical analysis conducted**

**Statistical analysis**

All downstream analyses were conducted in R v4.1.0 (R Core Team, 2023). Prior to permutational multivariate analysis of variance tests (PERMANOVA) homogeneity of multivariate dispersions was tested with the betadisper function in the vegan package v2.6 (Anderson, 2006) and raw count data was normalised using Hellinger transformation. Differences in community composition were assessed separately for rhizosphere and bulk soil samples using permutational multivariate analysis of variance (PERMANOVA) implemented in the adonis2 function from the vegan R package (v2.6). For each model, AMF (CTL, DAOM, C2) and microbial inoculum type (CTL, A, F) were included as predictor variables. All community dissimilarities were visualised using a non-metric multidimensional scaling (NMDS) ordination as implemented in phyloseq v1.38 R package. Pairwise comparisons were conducted using the adonis.pair function from the EcolUtils R package (v. 0.1). *P* values were corrected for multiple comparisons using the Holm method. Alpha diversity metrics were calculated, and significant differences were assessed with ANOVA. The full experimental design included 45 plants, with 5 biological replicates per treatment combination (AMF and microbial inocula).

Threshold Indicator Taxa Analysis (TITAN2;(Baker et al., 2023)) was applied to relate AMF colonisation percentage to bacterial taxa (amplicon sequencing counts) following standard protocols (Baker & King, 2010) by combining the samples from the two AMF treatments (DAOM and C2) and running separately for each inocula treatment (CTL, A and F). Prior to running TITAN2, the amplicon sequencing data counts were normalised using the Hellinger transformation. TITAN2 was also used to relate AMF colonisation to fungal taxa (Hellinger transformed amplicon sequencing counts). The genus *Rhizophagus* was the only fungal indicator taxa and came from our AMF inocula.

Given, PERMANOVA showed no statistically significant differences attributable to soil inoculation and TITAN2 found consistent bacterial taxa to be associated with levels of AMF colonisation regardless of soil inoculation soil inoculation replicates were pooled to give 15 replicates per AMF treatment. Differential expression analysis (DESeq2, v1.34.0) following the standard protocol (Love et al., 2014) was used to establish which microbial genes were upregulated or downregulated in the presence of AMF genotype DAOM or C2 versus the CTL (15 replicates for each AMF treatment based on pooling the soil inoculation treatments). Additionally, (DESeq2, v1.34.0) was used to establish which maize genes were upregulated or downregulated in each of the AMF genotype treatments (DAOM and C2) versus the control without AMF.

To investigate interactions between upregulated soil microbial genes, upregulated maize genes and AMF genes, we performed separate co-occurrence network analyses for each AMF genotype using igraph (v2.1.2) in R. For DAOM, there were 39 upregulated maize genes and 18 upregulated soil microbial genes and for C2 there were 24 upregulated soil microbial genes and 35 upregulated maize genes compared to controls with no AMF. All AMF genes with read alignments were included in the analysis, 4826 and 3910 genes for DAOM and C2 respectively. Pearson correlation coefficients were calculated between genes, and a threshold of 0.75 defined edges in the undirected, weighted network. Community detection was performed with the Louvain algorithm, and node-level metrics (degree, average path length, clustering coefficient, betweenness centrality) were calculated. Differences in these metrics between clusters were evaluated using one-way ANOVA. ANOVA tests revealed highly significant between-cluster differences across all node-level metrics (degree: F = 1154, P < .000; average path length: F = 3015, P < .000; clustering coefficient: F = 217, P < .000; betweenness centrality: F = 8.002, P < .000). For the C2 genotype, cluster membership also significantly affected all node level metrics degree (F = 807, P < .001), average path length (F = 4, P < .001), clustering coefficient (F = 146.1, P < .001), and betweenness centrality (F = 4.645, P = .00033). To create simplified plots a subnetwork comprising only genes from multi-kingdom clusters was extracted. AMF connector genes were defined as AMF-derived nodes with direct edges to both maize and microbiome genes. Connector hubs were subsequently identified as AMF connector genes with degree values greater than or equal to the 90th percentile of the connector degree distribution. A reduced hub-centred subnetwork was generated by retaining AMF connector hubs and their direct maize and microbiome neighbours for each AMF genotype.

**References for Supplementary Notes**

Abarenkov, K., Nilsson, R. H., Larsson, K.-H., Taylor, A. F. S., May, T. W., Frøslev, T. G., Pawlowska, J., Lindahl, B., Põldmaa, K., Truong, C., Vu, D., Hosoya, T., Niskanen, T., Piirmann, T., Ivanov, F., Zirk, A., Peterson, M., Cheeke, T. E., Ishigami, Y., … Kõljalg, U. (2024). The UNITE database for molecular identification and taxonomic communication of fungi and other eukaryotes: Sequences, taxa and classifications reconsidered. *Nucleic Acids Research*, *52*(D1), D791–D797. https://doi.org/10.1093/nar/gkad1039

Anderson, M. J. (2006). Distance‐Based Tests for Homogeneity of Multivariate Dispersions. *Biometrics*, *62*(1), 245–253. https://doi.org/10.1111/j.1541-0420.2005.00440.x

Baker, M. E., Kahle, D., & King, R. S. (2023). *TITAN2: Threshold Indicator Taxa Analysis* (Version 2.4.3) [Computer software]. https://CRAN.R-project.org/package=TITAN2

Baker, M. E., & King, R. S. (2010). A new method for detecting and interpreting biodiversity and ecological community thresholds: *Threshold Indicator Taxa ANalysis (TITAN)*. *Methods in Ecology and Evolution*, *1*(1), 25–37. https://doi.org/10.1111/j.2041-210X.2009.00007.x

Bolyen, E., Rideout, J. R., Dillon, M. R., Bokulich, N. A., Abnet, C. C., Al-Ghalith, G. A., Alexander, H., Alm, E. J., Arumugam, M., Asnicar, F., Bai, Y., Bisanz, J. E., Bittinger, K., Brejnrod, A., Brislawn, C. J., Brown, C. T., Callahan, B. J., Caraballo-Rodríguez, A. M., Chase, J., … Caporaso, J. G. (2019). Reproducible, interactive, scalable and extensible microbiome data science using QIIME 2. *Nature Biotechnology*, *37*(8), 852–857. https://doi.org/10.1038/s41587-019-0209-9

Buchfink, B., Xie, C., & Huson, D. H. (2015). Fast and sensitive protein alignment using DIAMOND. *Nature Methods*, *12*(1), 59–60. https://doi.org/10.1038/nmeth.3176

Callahan, B. J., McMurdie, P. J., Rosen, M. J., Han, A. W., Johnson, A. J. A., & Holmes, S. P. (2016). DADA2: High-resolution sample inference from Illumina amplicon data. *Nature Methods*, *13*(7), 581–583. https://doi.org/10.1038/nmeth.3869

Dobin, A., Davis, C. A., Schlesinger, F., Drenkow, J., Zaleski, C., Jha, S., Batut, P., Chaisson, M., & Gingeras, T. R. (2013). STAR: Ultrafast universal RNA-seq aligner. *Bioinformatics*, *29*(1), 15–21. https://doi.org/10.1093/bioinformatics/bts635

Glöckner, F. O., Yilmaz, P., Quast, C., Gerken, J., Beccati, A., Ciuprina, A., Bruns, G., Yarza, P., Peplies, J., Westram, R., & Ludwig, W. (2017). 25 years of serving the community with ribosomal RNA gene reference databases and tools. *Journal of Biotechnology*, *261*, 169–176. https://doi.org/10.1016/j.jbiotec.2017.06.1198

Ihrmark, K., Bödeker, I. T. M., Cruz-Martinez, K., Friberg, H., Kubartova, A., Schenck, J., Strid, Y., Stenlid, J., Brandström-Durling, M., Clemmensen, K. E., & Lindahl, B. D. (2012). New primers to amplify the fungal ITS2 region—Evaluation by 454-sequencing of artificial and natural communities. *FEMS Microbiology Ecology*, *82*(3), 666–677. https://doi.org/10.1111/j.1574-6941.2012.01437.x

Jiao, Y., Peluso, P., Shi, J., Liang, T., Stitzer, M. C., Wang, B., Campbell, M. S., Stein, J. C., Wei, X., Chin, C.-S., Guill, K., Regulski, M., Kumari, S., Olson, A., Gent, J., Schneider, K. L., Wolfgruber, T. K., May, M. R., Springer, N. M., … Ware, D. (2017). Improved maize reference genome with single-molecule technologies. *Nature*, *546*(7659), 524–527. https://doi.org/10.1038/nature22971

Kopylova, E., Noé, L., & Touzet, H. (2012). SortMeRNA: Fast and accurate filtering of ribosomal RNAs in metatranscriptomic data. *Bioinformatics*, *28*(24), 3211–3217. https://doi.org/10.1093/bioinformatics/bts611

Liao, Y., Smyth, G. K., & Shi, W. (2014). featureCounts: An efficient general purpose program for assigning sequence reads to genomic features. *Bioinformatics*, *30*(7), 923–930. https://doi.org/10.1093/bioinformatics/btt656

Love, M. I., Huber, W., & Anders, S. (2014). Moderated estimation of fold change and dispersion for RNA-seq data with DESeq2. *Genome Biology*, *15*(12), 550. https://doi.org/10.1186/s13059-014-0550-8

Manni, M., Berkeley, M. R., Seppey, M., & Zdobnov, E. M. (2021). BUSCO: Assessing Genomic Data Quality and Beyond. *Current Protocols*, *1*(12), e323. https://doi.org/10.1002/cpz1.323

Martin, M. (2011). Cutadapt removes adapter sequences from high-throughput sequencing reads. *Vol. 17(1):10*, *17*(1), 10–12. https://doi.org/10.14806/ej.17.1.200

McMurdie, P. J., & Holmes, S. (2013). phyloseq: An R Package for Reproducible Interactive Analysis and Graphics of Microbiome Census Data. *PLoS ONE*, *8*(4), e61217. https://doi.org/10.1371/journal.pone.0061217

Nossa, C. W. (2010). Design of 16S rRNA gene primers for 454 pyrosequencing of the human foregut microbiome. *World Journal of Gastroenterology*, *16*(33), 4135. https://doi.org/10.3748/wjg.v16.i33.4135

Quast, C., Pruesse, E., Yilmaz, P., Gerken, J., Schweer, T., Yarza, P., Peplies, J., & Glöckner, F. O. (2012). The SILVA ribosomal RNA gene database project: Improved data processing and web-based tools. *Nucleic Acids Research*, *41*(D1), D590–D596. https://doi.org/10.1093/nar/gks1219

R Core Team. (2023). *R: A language and Environment for Statistical Computing* [Computer software]. R Foundation for Statistical Computing. https://www.R-project.org/

Vierheilig, H., Coughlan, A. P., Wyss, U., & Piché, Y. (1998). Ink and Vinegar, a Simple Staining Technique for Arbuscular-Mycorrhizal Fungi. *Applied and Environmental Microbiology*, *64*(12), 5004–5007.

Wagner, M. R., Lundberg, D. S., Coleman-Derr, D., Tringe, S. G., Dangl, J. L., & Mitchell-Olds, T. (2014). Natural soil microbes alter flowering phenology and the intensity of selection on flowering time in a wild Arabidopsis relative. *Ecology Letters*, *17*(6), 717–726. https://doi.org/10.1111/ele.12276

White, T. J., Bruns, T., Lee, S., & Taylor, J. (1990). Amplification and direct sequencing of fungal ribosomal RNA genes for phylogenetics. In *PCR Protocols* (pp. 315–322). Elsevier. https://doi.org/10.1016/B978-0-12-372180-8.50042-1

Yilmaz, P., Parfrey, L. W., Yarza, P., Gerken, J., Pruesse, E., Quast, C., Schweer, T., Peplies, J., Ludwig, W., & Glöckner, F. O. (2014). The SILVA and “All-species Living Tree Project (LTP)” taxonomic frameworks. *Nucleic Acids Research*, *42*(D1), D643–D648. https://doi.org/10.1093/nar/gkt1209

Zhang, J., Kobert, K., Flouri, T., & Stamatakis, A. (2014). PEAR: A fast and accurate Illumina Paired-End reAd mergeR. *Bioinformatics*, *30*(5), 614–620. https://doi.org/10.1093/bioinformatics/btt593

**Supplementary Figure S1 – Relative abundances of bacterial and fungal taxa used for soil A and F inoculums**

(A) Relative abundance bar plot of bacterial taxa from the original soils and soil wash inoculums used to inoculate the A and F communities. (B) Relative abundance bar plot of fungal taxa from the original soils and soil wash inoculums used to inoculate the A and F communities.

**Supplementary Figure S2 – Taxonomic composition of bacteria in the rhizosphere and bulk soil**

(A) and (B) Non-metric multidimensional scaling (NMDS) ordination plots showing community structure of bacterial taxa in the rhizosphere (A) and bulk soil (B). PERMANOVA found no statistically significant differences in the composition of taxa due to AMF inoculation or inoculation with A or F communities. (C) and (D) Alpha diversity indices of soil bacterial communities in the rhizosphere (C) and bulk soil (D). No statistically significant differences were found between treatments.

**Supplementary Figure S3 – Taxonomic composition and diversity of fungi in the rhizosphere and bulk soil**

(A) and (B) Non-metric multidimensional scaling (NMDS) ordination plots showing community structure of fungal taxa in the rhizosphere (A) and bulk soil (B). PERMANOVA found no statistically significant differences in the composition of taxa due to AMF inoculation or inoculation with A or F communities. (C) and (D) Alpha diversity indices of soil fungal communities in the rhizosphere (C) and bulk soil (D). No statistically significant differences were found between treatments.
